# Bioactive Compounds from Cocoa Husk: Extraction, Analysis and Applications in Food Production Chain

**DOI:** 10.3390/foods11060798

**Published:** 2022-03-10

**Authors:** Tarun Belwal, Christian Cravotto, Sudipta Ramola, Monika Thakur, Farid Chemat, Giancarlo Cravotto

**Affiliations:** 1 Department of Drug Science and Technology, University of Turin, Via P. Giuria 9, 10125 Turin, Italy; tarungbpihed@gmail.com; 2 GREEN Extraction Team, INRAE, UMR 408, Avignon University, F-84000 Avignon, France; christian.cravotto@alumni.univ-avignon.fr (C.C.); farid.chemat@univ-avignon.fr (F.C.); 3 Research Group for Advanced Materials & Sustainable Catalysis (AMSC), State Key Laboratory Breeding Base of Green Chemistry-Synthesis Technology, College of Chemical Engineering, Zhejiang University of Technology, Hangzhou 310014, China; siramola@gmail.com; 4 Amity Institute of Food Technology, Amity University, Noida 201303, India; mthakur1@amity.edu; 5 World-Class Research Center “Digital Biodesign and Personalized Healthcare”, Sechenov First Moscow State Medical University, 119146 Moscow, Russia

**Keywords:** cocoa husk, extraction and analytical methods, functional foods, bioactive compounds, circular economy

## Abstract

Cocoa husk is considered a waste product after cocoa processing and creates environmental issues. These waste products are rich in polyphenols, methylxanthine, dietary fibers, and phytosterols, which can be extracted and utilized in various food and health products. Cocoa beans represent only 32–34% of fruit weight. Various extraction methods were implemented for the preparation of extracts and/or the recovery of bioactive compounds. Besides conventional extraction methods, various studies have been conducted using advanced extraction methods, including microwave-assisted extraction (MAE), ultrasonic-assisted extraction (UAE), subcritical water extraction (SWE), supercritical fluid extraction (SFE), and pressurized liquid extraction (PLE). To include cocoa husk waste products or extracts in different food products, various functional foods such as bakery products, jam, chocolate, beverage, and sausage were prepared. This review mainly focused on the composition and functional characteristics of cocoa husk waste products and their utilization in different food products. Moreover, recommendations were made for the complete utilization of these waste products and their involvement in the circular economy.

## 1. Introduction

Cocoa is one of the most utilized plant products in chocolates, snacks, and beverages. The annual production of cocoa was about 4.72 million tons in the year 2020, with an expected sale of around USD 20 billion in 2021 [1]. By the year 2025, the cocoa market is expected to grow at a compound annual growth rate (CAGR) of 7.33%. During cocoa processing, various byproducts including cocoa bean husk, cocoa shell, and pulp are generated, which is estimated to be 85% of the cocoa production [2]. If left behind, these waste products create pollution and economical losses [3]. The environmental impact of cocoa pod waste is related to methane and carbon dioxide generation by bacterial degradation; moreover, disposed of by-products can propagate diseases causing significant crop losses. The cocoa industry produces waste which causes environmental and ecological issues and which poses challenges for its proper utilization into the circular economy [4]. Unfortunately, considering the growing demand and utilization of cocoa products, this waste is generated at a larger proportion.

The cocoa waste products have been investigated and found to contain various bioactive compounds, including dietary fiber, polyphenols, pectin, methylxanthine, fat, and phytosterols [5]. The cocoa waste extract/fractions were tested and found effective in mitigating several disease conditions, including diabetes, hypercholesterolemia, hypertension, and inflammation [6]. To improve the extract quality, various advanced extraction methods were carried out in the last decades. These mainly include, ultrasonics, supercritical CO_2_, microwaves, pressurized liquid extraction, and subcritical water extraction. Moreover, efforts have been made to create greener processes by replacing organic solvents with green solvents (deep eutectic solvents, water, CO_2_). The cocoa waste extract/powder was also utilized to add value to various food products (cake, chocolate, beverages, etc.) and improve their physicochemical and functional properties. To utilize these cocoa waste products in a green circular economy concept, a greener processing strategy is expected. The development of greener technology allows for the recovery of valuable compounds from these wastes in a green and sustainable manner, thereby providing a direct way to utilize them in food products.

Over the last decade, several investigations have been conducted to extract all the bioactive compounds to be applied in food products [4]. The present review will discuss the recent advances in the extraction of bioactive compounds and their utilization in the food production chain. Moreover, the recommendations were made on the complete utilization of cocoa waste (as a raw material and after processing), which could be adopted for the circular economy concept.

## 2. Bioactive Compounds Extraction from Cocoa Bean Husk

**Figure 1:**
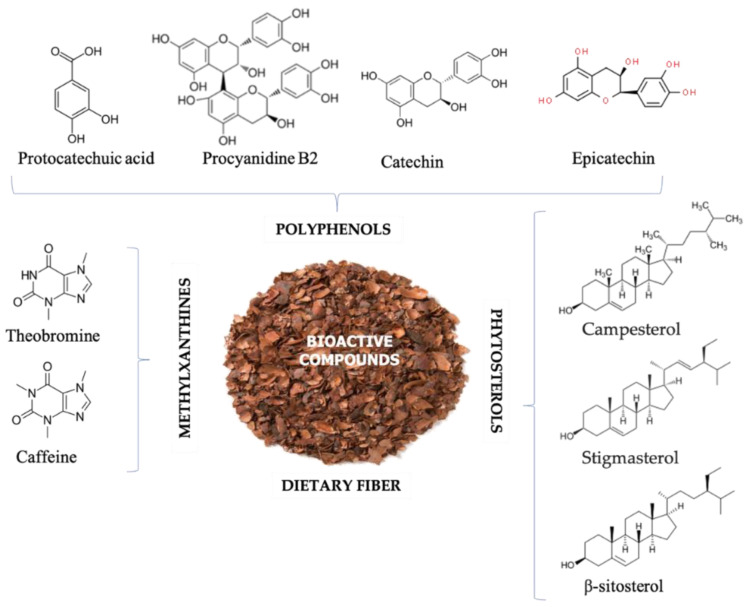
Major bioactive compounds extracted from cocoa bean husk and cocoa shell.

Waste material generated during production includes cocoa pod husk, pulp, and cocoa bean shells. Dietary fiber, polyphenols, methylxanthines, phytosterols are some of the major compounds extracted from cocoa pod husks or cocoa shells (Figure 1). In recent decades, these compounds have been extracted using advanced/green extraction technologies and tested for their application in food products (Table 1).

Pectins are natural polymers used for a variety of applications, including emulsifiers, gelling agents, thickeners, stabilizers, and fat or sugar replacers [30]. In a study, pectin from cocoa pod husk was analyzed to determine its physicochemical and antimicrobial activity [7]. The pectin was extracted using aqueous citric acid (4% *w*/*v*) followed by precipitation of extract using ethanol. A pectin yield of 23.3% was obtained with a degree of esterification of 26.8% and showed good physicochemical properties. The pectin was characterized as highly acetylated low-methoxy pectin having rich mineral contents. The obtained product also showed high antimicrobial activity against *E. coli* and *S. aureus*. In another study, the effect of extraction conditions on pectin yield from cocoa husk was investigated [8]. Various solvents including water, citric acid (pH 2.5, 4), and hydrochloric acid (pH 2.5, 4) were investigated. It was found that temperature, time, and sample-to-solvent ratio affected the pectin yield. As such, the highest yield of pectin (7.62%) was obtained using citric acid (pH 2.5) as a solvent, an extraction temperature of 95 °C for 3 h, and a sample-to-solvent ratio of 1:25.

Citric acid is mostly used for extracting pectin. However, the use of nitric acid, oxalic acid, and ascorbic acid-based extraction of pectin from cocoa pod husk has also been reported. As such, using nitric acid-based extraction of pectin from cocoa pod husk, it was found that increasing the temperature increased the extraction yield [9]. The optimum extraction condition was a pH of 1.5, extraction temperature of 100 °C, and extraction time of 30 min. Oxalic acid-based extraction under microwave radiation conditions was intended to separate pectin from cocoa pod husk. It was found that a higher oxalic acid concentration, lower pH, and irradiation time increased pectin yield. Moreover, under microwave conditions, the extraction time was shorter than the conventional extraction [10]. Ascorbic acid-based extraction of pectin from cocoa pod husk was carried out. The optimum condition was pH 2.5, 95 °C, for 45 min of extraction time [11].

### 2.1. Microwave-Assisted Extraction

The use of microwave technology for extracting bioactive compounds has been tremendously increased due to its commercial applications and advances [31]. As such, the use of microwave technology for extracting bioactive compounds from cocoa husk pods has also been investigated over the last two decades. In another study, microwave-assisted extraction (MAE) of the cocoa bean shell was carried out to obtain polyphenol and polysaccharide fractions which were used to produce pectin-based films [19]. The film was prepared using pectin and cocoa bean shells, and ZnO/Zn nanoparticles were added to improve the thermal, barrier, structural, morphological, and optical properties of the film. The obtained biofilm prepared by the pectin–cocoa bean shell extract–ZnO/Zn nanoparticles showed greater UV and oxygen barrier properties and thus can be used for active packaging for increasing the shelf-life of food products.

Anthocyanins are polyphenolic compounds known for their color properties and health effects [32]. Anthocyanin was extracted from cocoa peel using MAE and factors such as extraction time, microwave power, particle size, and sample-to-solvent ratio were investigated using response surface methodology [20]. The highest yield of anthocyanin obtained from MAE was 1.435 mM under the optimum condition of particle size (60 mesh), sample-to-solvent ratio (0.0625 *w*/*v*), extraction time (10 min), and microwave power (450 W).

Phytosterols play an important role in maintaining health and mitigating several disease conditions [33]. As such, their extraction from plants has been largely focused on obtaining and utilizing them for various health products [34]. In an attempt, the optimum extraction conditions for β-sitosterol (a phytosterol) from cocoa shells were determined under MAE conditions using absolute ethanol [21]. The effect of temperature, microwave power, and radiation time was determined, and the optimum extraction condition was recorded as 70 °C, 500 W, and 10 min, respectively. The β-sitosterol yield under optimum extraction conditions was recoded as 3546.1 mg/100 g, which was 13% higher than the yield obtained using the conventional maceration. Catechin was also extracted from cocoa husk waste using MAE [17]. The MAE temperature was set at 70 °C, and the cocoa waste was extracted with ethanol at a ratio of 3:100 g/mL under varying extraction times (4, 6, 8, and 10 min). It was found that a longer extraction time of 8 and 10 min produces a higher extraction yield of total phenolic content and total catechin content.

The phenolic antioxidant compounds extracted under MAE conditions showed high antioxidant activity [19]. The optimum extraction condition was an extraction time of 5 min, pH of 12, a temperature of 97 °C, and a sample-to-solvent ratio of 0.04 g/L. Moreover, pH played an important role and an alkaline pH promoted the extraction of compounds. At a high pH value, the extract was rich in protein, polysaccharides, and polyphenols, and had high antioxidant activity.

### 2.2. Water Extraction

Green extraction technology with minimum utilization of energy, time, resources, and free from organic solvents is considered an environmentally friendly and sustainable solution [35]. Green extraction of phenolics from cocoa husk powder using water as a solvent was carried out using heat-assisted extraction [16]. Factors such as extraction temperature, time, acidity, and sample-to-solvent ratio were optimized. The highest extraction yield for total phenolic compounds, total flavonoids, total flavanols, total phenolic acids, total proanthocyanidins, total ortho-diphenols, and antioxidant activity was recorded at 100 °C, 90 min, 0% citric acid, and 0.02 g cocoa shell/mL of water. The compounds reported in the extract are hydroxybenzoic acid, hydroxycinnamic acid, mandelic acid, phenylacetic acid, flavan-3-ols (monomers/dimers), flavonols. Moreover, the water extract was compared with the organic extract, and it was found that most of the compounds were extracted in the water. As such, quercetin 3-O galactoside, quercetin 3-O-glucoside, procyanidin B2, procyanidin B1, (+)- catechin, and (−)-epicatechin were recorded in higher concentrations. In addition, compounds such as mandelic acid, 3,4-dihydroxyphenylacetic acid, and 4-hydroxyphenylacetic acid were recorded only in the water extract.

### 2.3. Extraction in Supercritical CO_2_

Fat and methylxanthines (theobromine and caffeine) were extracted from cocoa shells using supercritical CO_2_ [23]. Pressure (2000–6000 psi), temperature (313–333 K), and time (30–90 min) were varied to obtain the optimum extraction condition. It was found that the fat yield is around 94.73% (which is the most effective extraction), while for caffeine the extraction yield is about 90%; however, theobromine could not be extracted under optimum supercritical CO_2_ extraction conditions (6000 psi, 313 K, 90 min) due to low solubility.

Dietary fiber plays an important role in human health and metabolism [36] and was extracted from cocoa shells using high voltage electric discharge conditions [24]. It was found that the high-voltage electrical discharge condition had a significant impact on the physical properties of the dietary fiber. Furthermore, it increased the fiber content, grinding ability, and water-binding capacity. It was also observed that the tannin content changed during the high voltage discharge treatment, which had a significant impact on the fiber pretreatment and resulted in a more undigested sample. Supercritical fluid extraction was conducted to extract polyphenols from the cocoa husk. The particle size, extraction temperature, time, pressure, and ethanol concentration were optimized. The results showed a particle size of less than 0.26 mm, extraction time of 147 min, extraction temperature of 308.15 K, and pressure of 20 MPa; moreover, 20% of ethanol increased TPC, total flavon-3-oles, and total carotenoids content [13]. Supercritical CO_2_ extraction was found to increase the phenolic compounds’ extraction and antioxidant activity using ethanol as a co-solvent under the optimal extraction condition of 60 °C, 299 bar, and 13.7% ethanol concentration [37]. The combined effect of supercritical fluid extraction and pressurized liquid extraction (using ethanol) on the antioxidant compounds’ extraction from cocoa bean hulls was investigated. It was found that a higher phenolic content and antioxidant activity were recorded under the combined extraction process compared to the individual extraction process [29].

### 2.4. Subcritical Water Extraction

Subcritical water extraction is one of the recent advances in extraction technologies and is considered green [38]. It changes the properties of water by varying the temperature and pressure, thereby affecting solubility, mass transfer, and extraction capacity. Various factors, such as temperature, time, and sample to solvent ratio were varied from 120–220 °C, 15–75 min, and 1:10–1:30 g/mL to obtain very high-end products [25]. Various compounds were detected under optimum extraction conditions (temperature 170 °C, time 75 min, sample to solvent ratio 1:20). These include theobromine, caffeine, theophylline, gallic acid, epicatechin, catechin, chlorogenic acid, and total phenols. Moreover, other chemical molecules, such as mannose, glucose, xylose, arabinose, rhamnose, and fructose, and 5-hydroxy methylfurfural, furfural, levulinic acid, and formic acid were detected. In another study, phenolics from cocoa bean shell was extracted using subcritical water and then encapsulated with maltodextrin and whey protein using a spray drying technique [26]. The SWE was carried out at a temperature of 150 °C with an extraction pressure of 30 bar for 15 min. It was found that whey protein protects the phenolic content resulting in a higher content of gallic acid, caffeine, and theobromine as compared to maltodextrin. Using subcritical water extraction technology, pectin was extracted from cocoa pod husk at a higher yield as compared to citric acid-based extraction [15].

### 2.5. Ultrasound-Assisted Extraction

Ultrasound-assisted extraction of flavonoids from cocoa shells was introduced to determine the optimum extraction conditions [22]. Ethanol concentration (70–90%), temperature (45–65 °C), and irradiation time (30–60 min) were optimized. The highest total flavonoid yield was obtained as 7.47 mg RE/g dw at 80% ethanol, 55 °C, and 45 min of time.

The pressurized liquid extraction method was used to obtain the ethanolic extract of cocoa bean shells containing flavonoids and alkaloids. With variation in temperature and extraction time, the extraction yield of the compounds was affected. As such, by increasing the extraction temperature and time, the flavonoid and alkaloid extraction increased, while the procyanidins B2 degraded. It was interesting to note that the lyophilized extract showed higher flavonoids (catechin, epicatechin, procyanidin B2) and alkaloid (theobromine, caffeine) content as compared to the dried cocoa shell powder extract [18].

### 2.6. Conditions and Solvents Optimization

The particle size of a plant sample for extraction is usually ignored. In one study, the impact of cocoa bean shell particle size was tested on the physicochemical, bioactive compounds, and antioxidant activity [28]. Three particle sizes were considered, i.e., high (Dp > 701 um), intermediate (417 um< Dp < 701 um), and lowest (Dp < 417 um). It was found that as the particle size reduced, the extraction efficiency for dietary fiber (65.58 g/100 g), polyphenolic compounds (epicatechin, 6.33 mg/g; catechin, 4.58 mg/g), and methylxanthine (theobromine, 12.77 mg/g; caffeine, 6.13 mg/g) was increased.

The effect of solvents on the extraction of bioactive compounds from cocoa waste was also studied [12]. In an attempt, the theobromine-rich extract was prepared by varying the solvents (water, chloroform, and 70% ethanol), extraction time (30, 60, and 90 min), and the number of extraction cycles (1 or 2). The optimal extraction condition for the maximum theobromine yield (6.79 mg/100 g) was found as 70% ethanol, extraction time of 90 min, temperature of 80 °C, and 1 cycle of extraction. Alcoholic solvents under atmospheric pressure were used to extract the bioactive compounds and fat content. Two solvents, ethanol and isopropanol, were used at 75 °C and 90 °C, respectively. The fat content was obtained in the range of 3–70% with absolute solvents. Hydrated alcohol was found to be suitable for extracting bioactive compounds, especially for alkaloids (73% yield) [39]. In another study, the different phytochemicals from the cocoa husk and cocoa bean were analyzed [40]. In the cocoa husk, a high content of phenolic acid was recorded, while in the cocoa bean, a high content of flavonoids was recorded. A total of 49 compounds were detected.

Deep eutectic solvent (DES) is a new class of green, non-flammable solvents typically formed by mixing choline chloride with hydrogen bond donors [41]. Deep eutectic solvents (DESs) have been used to extract bioactive compounds from cocoa shells. In one study, MAE was performed using DES for extracting bioactive compounds from cocoa shells [27]. The yield of the compounds in DES was lower than DES/MAE. For instance, the yield of theobromine was 2.5–5.0 mg/g under DES/MAE, while under DES it was 2.1 to 4.6 mg/g. Similarly, for caffeine, it was 0.778–1.599 mg/g in DES/MAE, while it was 0.68–1.52 mg/g under DES; however, the DPPH antioxidant activity was lower in DES/MAE compared to DES. It was found that the water content in different choline chloride-based DES influences the oxidation, while the extraction time and temperature showed no significant impact. Heat-stirring assisted extraction (HSE) or ultrasound probe-assisted extraction was used along with deep eutectic solvents for preparing extracts rich in phenolics and alkaloids [14]. It was found that ultrasound (3 min, 200 W) DES (lactic acid:ChCl) was superior in extracting the compounds (chlorogenic acid, caffeine, and theobromine) compared to HSE.

## 3. Functional Food Containing Cocoa Husk Powder/Extract

**Figure 2:**
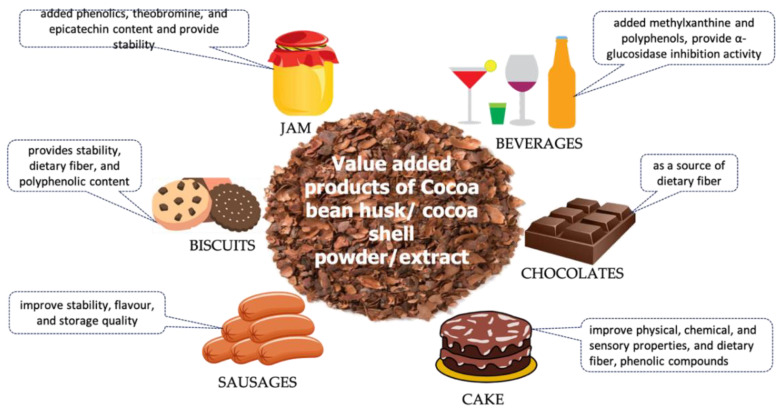
Valorization of food products with cocoa bean husk or cocoa shell extract.

Being a rich source of bioactive compounds, the cocoa husk powder/extract has been tested and used as an additive in various food products for improving the physical, chemical, and biological properties of the products [42]. These bioactive compounds showed remarkable biological activities, which could provide functionality to food products (Figure 2).

The dietary fiber from cocoa bean husk was found effective against various disease conditions. As such, soluble dietary fiber, insoluble dietary fiber, and total dietary fiber were produced from cocoa bean shells and tested for hypoglycemic and cholesterol-lowering effects. Among all, soluble dietary fiber showed higher glucose adsorption capacity, α- amylase inhibition activity, cholesterol, and sodium cholate binding capacity [43]. Moreover, cocoa shell flour was found to prevent hyperlipidemia in HepG2 cells [44]. Due to the presence of a high quantity of dietary fiber, cocoa bean husk was used in several food formulations. Chocolate, as such, is considered as having nutritional and caloric value; however, the addition of fiber further improved the nutritional properties, while decreasing the polyphenolic content. In one study, cocoa shell (as a source of dietary fiber) was added to dark and milk chocolate and improved the dietary fiber content without any major impact on the polyphenolic content, and hence could be considered for its use. The quality of the prepared chocolate was comparable to commercial chocolate [45]. Cocoa hull phenolic extract was prepared and encapsulated using spray drying, incorporated in biscuits preparation, and tested for its stability during the baking process. Polyphenols were extracted using ethanol under ambient temperature for 30 min. The spray-dried powder was used to produce biscuits containing wheat flour (55.2%), sugar (13.7%), shortening (29.5%), and cocoa spray-dried powder (0.32%). Microencapsulation improves the stability of polyphenolic compounds in biscuits [46]. In a similar study, high-fiber functional biscuits were prepared using cocoa bean shells and tested for their consumption by diabetic patients. The biscuits showed α-glucosidase inhibitory activity [47]. In a cake formation, vegetable oil was substituted with cocoa bean hull by 30, 40, and 50%, and was found to effectively improve the physical, chemical, and sensory properties. It was found that the cocoa hull cake increased dietary fiber, phenolic compounds, and antioxidant activity [48]. The dietary fiber properties of cocoa husk were tested for their influence on physicochemical and sensory properties of emulsion-type pork sausages at different concentrations of cocoa powder (0.25–2%). It was found that cocoa powder increased the stability of the emulsion, increased flavor acceptability, and overall product acceptability. Furthermore, it significantly inhibited lipid peroxidation in the sausages during storage (refrigerated) [49].

The polyphenols and methylxanthine compounds in cocoa bean shell extract exerted various biological activities. As such, the antioxidant and apoptotic activity of cocoa husk extract was reported when tested on prostate cancer cells. The fraction (ethylacetate and butanol) of the extract was found to contain catechin, epicatechin, and procyanidin B. It was found that the extract showed antioxidant and apoptotic activity in PC3 and DU145 cells [50]. In another study, the anti-hypertensive and anti-hyperuricemia effects of cocoa pod husk extract were reported by inhibiting xanthine oxidase and angiotensin-1-converting enzyme and scavenging free radicals [51]. In one study, the skin whitening effect of a cocoa pod extract was determined based on a tyrosinase assay and sun-screening effect (UV 200–400 nm). It showed inhibition of the tyrosine enzyme and exhibited a UV-B sunscreen effect, thereby exerting anti-wrinkle skin whitening and sunscreen effects [52]. Some studies also suggested the effect of cocoa husk extract in the treatment of oral cavities and related symptoms. As such, in one study, a cocoa bean husk extract was tested for its mouth rinse activity in children (10 mL of 0.1%) and was found to inhibit the *Streptococcus mutans* count in saliva; the results were comparable with chlorhexidine (commercial product) [53]. Root canal treatment failure is mostly due to the presence of *Enterococcus faecalis*. Cocoa pod husk extract was found effective against *E. faecalis* at an extract concentration of 3.12% [54]. Moreover, it was found that cocoa bean shell extract (with concentrated epicatechin and tannin) counteracted oxysterol-induced inflammation in vitro [55].

Due to the presence of polyphenols and methylxanthine content in cocoa bean husk extract, it has been tested in various food products for improving product functionality. As such, cocoa bean shell extract containing polyphenols and methylxanthine was added to a flavored beverage after in vitro digestion for improving its functionality and consumer acceptance. It was found that the bioaccessibility of methylxanthine was 100%, while for polyphenols (B procyanidins and epicatechin) it was 50%. An increased α-glucosidase inhibition activity of the value-added beverage was also recorded with high acceptability of the product by the consumer [56]. In another study, the utilization of cocoa bean husk extract (obtained by the thermal treatment at 170 °C for 30 min) into a virgin olive oil jam in freeze-dried form or encapsulated form was investigated. It was found that the cocoa extract provided stability to the product and contained value-added phenolics, theobromine, and epicatechin. In addition, it was found that in food rich in fat/oil, the lyophilized form of the cocoa extract was suitable, while for aqueous food products the encapsulated cocoa extract was more effective [57].

To increase in the bioactivity of the natural products the fermentation technology has been effectively used in recent years. As such, a solid-state fermentation was used to increase the bioactivity of cocoa pod husk using *Pleurotus ostreatus* or *Calocybe indica*. It was found that the obtained extract after fermentation showed higher antimicrobial activity against bacteria (*Bacillus cereus, Methicillin Resistant Staphylococcus aureus, Salmonella paratyphi, Pseudomonas aeruginosa, Escherichia coli*, and *Klebsiella pneumoniae*) and fungi (*Candida albicans, Aspergillus niger, A. favus*, and *Trichophyton rubrum*) as compared to non-fermented extract. The obtained phytochemicals in the extract included polyphenolics, glycerine, pimelic ketone, D-ribonic acid, methyl myristate, palmitic acid methyl ester, oleic acid ethyl ester, lauramide, oleic acid amide, 1,2-cyclododecanediol, resorcinol, phytol, and others [58]. In another solid-state fermentation study [59], the cocoa shell was used as a raw material and utilized by *Penicillium roqueforti* for its conversion into valuable products. It was found that after the fermentation, a significant increase in the phenolic compounds and total carotenoid concentration was recorded, while the concentrations of anthocyanins and flavonoids did not change significantly. Moreover, saponin concentration and antioxidant potential along with oleic, denoleic, linolenic, and saturated fatty acids were increased after the cocoa shell fermentation.

## 4. The Cocoa Pod Husk/Shell Solid Waste after Extraction: Circular Economy Concept?

**Figure 3:**
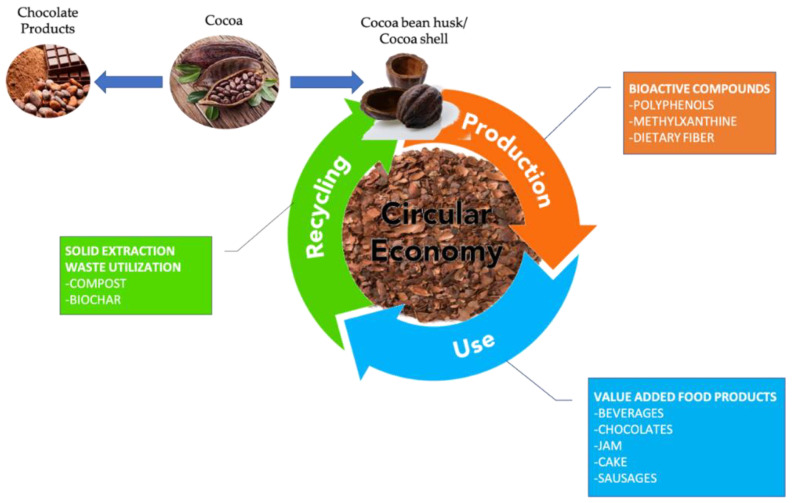
Circular economy concept for cocoa bean husk products. For the circular economy, the cocoa shell husk processed waste products (e.g., residue left after extraction of compounds) could be recycled/reused (e.g., biochar, compost) to provide a complete waste valorization solution with potential sustainable benefits.

The emerging new green extraction technologies (subcritical water extraction, supercritical fluid extraction) have provided an eco-friendly sustainable means of preparing and extracting the compounds from cocoa bean husk and cocoa shells. These green extraction processes utilize natural products without affecting the environment and make the process more efficient; however, in most cases, the solid residue remaining after the extraction process creates environmental problems. To meet the demands of the circular economy, not only does the food waste/byproducts utilization need to be conducted but the waste processing (e.g., solid residue after extraction) also needs to be utilized in a sustainable manner (Figure 3).

In the case of cocoa pod husk or cocoa shell extraction, the solid residue was left behind. These are mainly discarded as waste and cannot fully justify the circular economy concept. To date, only some research has been conducted utilizing this solid waste after extraction (SWE). Moreover, some research has been conducted to utilize the cocoa pod husk for preparing compost [60] and biochar [61,62], which was further used as a plant nutrient/fertilizer [63,64] and for the bioremediation of toxic chemicals from an aqueous medium [65,66]. In line with this, the SWE residues remaining after the extraction could be treated to remove chemical entities and then processed to form compost and biochar products for their complete utilization in a circular economy approach. Efficient industrial exploitation of the residual fibers is clearly expected; however, capital investment strongly depends on biomass fractions upgrading (lignin cellulose and hemicellulose). Despite the high content of lignin in cocoa bean shells and pod husks (15–39%) [4], its recovery from the product stream with high purity still remains a challenging task. Highly efficient processes for biomass fractionation and lignin depolymerization should be rapidly implemented in industrial biorefineries for the preparation of new materials from lignin monomers [67]. The relevant advances in circular economy strategies have prompted new business models supported by enabling technologies and operative skills that could reduce the costs of cocoa husk extracts. Purified extracts enriched in flavanols, flavonols, and other polyphenols showed impressive nutraceutical properties and value compared to the traditional management of cocoa husk as agricultural composting and mulching are one order of magnitude higher.

## 5. Conclusions

The present review highlighted advanced protocols and enabling technologies for extracting bioactive compounds from cocoa bean husk and cocoa shells over the last decade. The applications of cocoa by-products extract in different food products for improving physicochemical and functional properties were also reviewed.

Research on the extraction of bioactive compounds strongly suggests the use of advanced green extraction technologies for the recovery of pectin, dietary fibers, polyphenols, methylxanthine, and phytosterols. The use of microwave and supercritical CO_2_ are seen in a larger context, provided the application of these high-end technologies. Moreover, research on green extract preparation is gaining pace using subcritical water extraction and by replacing organic solvents with deep eutectic solvents. The extract has been used in various food products, including cake, chocolates, sausages, biscuits, jam, and beverages, and was found to improve physicochemical and functional properties. To utilize the growing cocoa waste production, the greener process is preferably used to not only limit the environmental runoff of hazardous chemicals but to also provide more economical, less laborious, and process-efficient products. The focus should also be on the zero waste/complete utilization of cocoa waste in food products and solid waste after extraction to biochar and compost for the circular economy and sustainability.

## Figures and Tables

**Table 1:** Extraction methods used for obtaining bioactive compounds from cocoa pod husk or cocoa shell in the last decade.

Compounds	Extraction Method	Yield (*w*/*w*)	References
**Cocoa pod husk**			
Pectin	Aqueous citric acid (4% *w*/*v*) followed by precipitation of extract using ethanol	23.3%	[7]
Pectin	Water, citric acid (2.5, 4 pH), and hydrochloric acid (2.5, 4 pH)	7.62%	[8]
Pectin	Nitric acid, pH 1.5, 100 °C of extraction temperature, and 30 min of extraction time	9.0%	[9]
Pectin	Oxalic acid + microwave radiation condition at pH 1.16, L/S = 25.0 and 15 min. of irradiation time	9.64%	[10]
Pectin	Ascorbic acid-based extraction, pH 2.5, 95 °C, for 45 min	4.2%	[11]
Theobromine rich extract	70% ethanol, extraction time of 90 min, temperature of 80 °C, and 1 cycle of extraction	Theobromine yield (6.79 mg/100 g)	[12]
TPC, total flavon-3-oles, and total carotenoids content	Supercritical fluid extraction, particle size less than 0.26 mm, extraction time of 147 min, extraction temperature of 308.15 K, pressure of 20 MPa, and 20% ethanol	TPC (35.11 EAG mg/g), a total flavan-3-oles content (12.89 EEP mg/g) and total carotenoids content (64.35 EBC mg/g)	[13]
phenolics and alkaloids	Heat-stirring assisted extraction (HSE) or ultrasound probe assisted extraction was used along with deep eutectic solvents	ultrasound (3 min, 200 W) Des (lactic acid:ChCl) was found superior in extracting the compounds (chlorogenic acid, caffeine, and theobromine) compared to HSE	[14]
Pectin	Subcritical water extraction	121 °C, 103.4 bar, and 30 min	[15]
Total phenolic compounds, total flavonoids, total flavanols, total phenolic acids, total proanthrocyanidins, total ortho-diphenols, and antioxidant activity	Heat-assisted extraction, 100 °C, 90 min, 0% citric acid, and 0.02 g cocoa shell/mL of water	UPLC-ESI-MS/MS revealed the presence of 15 phenolic compounds, being protocatechuic acid, procyanidin B2, (−)-epicatechin, and (+)-catechin, the major ones	[16]
Total phenolic content and total catechin content	MAE, absolute ethanol, 70 °C, 3:100 g/mL, 8 and 10 min	Total phenol content (TPC) and total catechin content (TCC)	[17]
**Cocoa bean shell**			
Flavonoids and alkaloids	Pressurized liquid extraction	Lyophilized extract showed higher flavonoids (catechin, epicatechin, procyanidin B2) and alkaloid (theobromine, caffeine) content as compared to the dried cocoa shell powder extract	[18]
Polyphenols and polysaccharides- pectin-based films	Microwave-assisted extraction (MAE)	obtained biofilm prepared by pectin-cocoa bean shell extract-ZnO/Zn nanoparticle showed greater UV and oxygen barrier properties	[19]
Anthocyanin	MAE, particle size (60 mesh), sample to solvent ratio (0.0625 *w*/*v*), extraction time (10 min), and microwave power (450 W)	1.435 mM	[20]
β-sitosterol	MAE, absolute ethanol, 70 °C, 500 W, and 10 min	3546.1 mg/100 g	[21]
Flavonoids	Ultrasound-assisted extraction under 80% ethanol, 55 °C, for 45 min	TFC = 7.47 mg RE/g dw	[22]
Protein, polysaccharide, and polyphenols	MAE, 5 min of extraction time, pH of 12, 97 °C of temperature, and sample to solvent ratio of 0.04 g/L	Pectin-based films	[19]
Fat and methylxanthines (theobromine and caffeine)	Supercritical CO_2_, 6000 psi, 313 K, 90 min	94.73% (which is most effective extraction), while for caffeine the extraction yield is about 90%	[23]
Dietary fiber	High-voltage electric discharge	Increased fiber content	[24]
Polyphenols and methylxanthines	Subcritical water extraction, temperature 170 °C, time 75 min, sample to solvent ratio 1:20	theobromine, caffeine, theophylline, gallic acid, epicatechin, catechin, chlorogenic acid, and total phenols	[25]
Polyphenols and methylxanthines	Subcritical water extraction, 150 °C with extraction pressure of 30 bar for 15 min	that whey protein protects the phenolic content resulted in higher content of gallic acid, caffeine, and theobromine as compared to maltodextrin	[26]
Alkaloids	MAE was performed using DES	Theobromine (2.502–5.004 mg/g) and caffeine (0.778–1.599 mg/g)	[27]
Dietary fiber, polyphenolic compounds, and methylxanthine	Particle sizes were considered, i.e., high (Dp > 701 um), intermediate (417 um < Dp < 701 um) and lowest (Dp < 417 um)	Dietary fiber (65.58 g/100 g), polyphenolic compounds (epicatechin, 6.33 mg/g; catechin, 4.58 mg/g), and methylxanthine (theobromine, 12.77 mg/g; caffeine, 6.13 mg/g)	[28]
Phenolics	Combined effect of supercritical fluid extraction and pressurized liquid extraction	TPC values from 35 to 51 mg GAE/g and EC50 values from 115 to 177 µg/mL	[29]

## Data Availability

Not applicable.
